# Dialysis Disequilibrium Syndrome and Severe Metabolic Acidosis: A Fatal Case

**DOI:** 10.7759/cureus.89369

**Published:** 2025-08-04

**Authors:** Armando F Rodriguez Lopez, Messanh K Ameduite, Veeranna Maddipati, Hunter Coore

**Affiliations:** 1 Nephrology, East Carolina University Health Medical Center, Greenville, USA; 2 Pulmonary and Critical Care, East Carolina University Health Medical Center/East Carolina University Brody School of Medicine, Greenville, USA; 3 Pulmonary and Critical Care, East Carolina University Brody School of Medicine, Greenville, USA; 4 Internal Medicine, East Carolina University Brody School of Medicine, Greenville, USA

**Keywords:** brain acidosis paradox, dialysis disequilibrium syndrome, fatal dialysis disequilibrium syndrome, severe metabolic acidosis, urea reverse phenomenon

## Abstract

Dialysis disequilibrium syndrome (DDS) is a rare but potentially fatal complication of renal replacement therapy, typically characterized by cerebral edema and often precipitated by the rapid correction of severe azotemia. Clinical symptoms are often non-specific, and, in some cases, the condition can be fatal. While the role of azotemia in DDS is well established, alternative mechanisms, such as the brain acidosis paradox, have also been proposed. We present the case of a 57-year-old African American woman with chronic kidney disease who was admitted with urosepsis and respiratory distress. Initial laboratory evaluation revealed a serum bicarbonate level <5 mEq/L and blood urea nitrogen of 116 mg/dL (baseline = 40-50 mg/dL). Due to the severity of her metabolic acidosis and signs of impending hypoxic respiratory failure, she underwent urgent hemodialysis. After 80 minutes of treatment, the patient became hypotensive and was transferred to the intensive care unit. Subsequent imaging revealed worsening cerebral edema with associated herniation, which was refractory to osmotherapy. The patient was transitioned to palliative care, and organ procurement was arranged. Most reported cases of fatal DDS are associated with chronic azotemia >150 mg/dL, a threshold not reached in this case. While preventive strategies for DDS emphasize limiting the urea reduction ratio (URR), there are currently no established guidelines for the safe rate of bicarbonate correction. In this case, the URR was 57% using a standard blood flow rate, and the bicarbonate level increased by more than 10 mEq/L. We hypothesize that rapid correction of metabolic acidosis may contribute to fatal DDS by worsening cerebral edema through mechanisms such as the brain acidosis paradox.

## Introduction

Dialysis disequilibrium syndrome (DDS) is a rare but serious complication of renal replacement therapy (RRT), characterized by cerebral edema. A review of fatal DDS cases frequently identifies severe azotemia and metabolic acidosis as key contributors [1-3]. Proposed mechanisms include the reverse urea osmotic gradient, the idiogenic osmole theory, and the paradoxical brain acidosis effect [4-7]. However, the potential cumulative or synergistic effect of these pathophysiologic processes in contributing to fatal outcomes remains underexplored. While there is no established consensus on an ideal urea reduction ratio (URR) to prevent DDS, clinical practice often employs a slow dialysis approach (blood flow rate of 150-250 mL/minute), targeting a URR below 45%, despite the absence of randomized controlled trial data [8-11]. Similarly, the optimal rate for correcting metabolic acidosis has not been clearly defined. This case highlights the potential temporal and mechanistic interplay between severe azotemia, rapid acidosis correction, and cerebral edema, supporting a contributory role of the brain acidosis paradox in the development of fatal DDS.

## Case presentation

A 57-year-old African American woman with a history of non-dialysis-dependent stage 4 chronic kidney disease (CKD) secondary to obstructive uropathy in the setting of a neurogenic bladder presented to the emergency department with six days of progressive shortness of breath, malaise, and generalized weakness. Her past medical history was significant for left renal atrophy, non-obstructive nephrolithiasis, chronic metabolic acidosis, anemia of CKD, and recurrent urinary tract infections with multidrug-resistant organisms. She also had a history of small bowel obstruction and endometriosis. Her surgical history included colostomy creation with subsequent reversal, total hysterectomy, bilateral nephrostomy tube placements, ureteral stent placement, and eventual permanent suprapubic catheter insertion. On a review of systems, she reported vague nausea without vomiting, decreased urine output despite adequate fluid intake, abdominal fullness and tenderness, serous discharge from her suprapubic catheter, increased respiratory effort, and trace pedal edema. She denied fever, chills, chest pain, flank pain, hematuria, skin rash, diarrhea, syncope, or appetite loss. Home medications included sodium bicarbonate 650 mg twice daily, ferrous sulfate 325 mg daily, potassium chloride 20 mEq three times daily, and docusate sodium 100 mg daily for constipation. Vital signs on presentation were temperature 37.2°C, respiratory rate 28 breaths per minute, heart rate 95 beats per minute, blood pressure 108/64 mmHg, and oxygen saturation 88% on room air. On physical examination, the patient appeared well-nourished but acutely ill. She was alert with a Glasgow Coma Scale score of 15/15. Her body mass index was 30.5 kg/m². She had moist oral mucosa, labored breathing, diffuse abdominal tenderness, foul-smelling suprapubic catheter discharge, and bilateral pitting pedal edema. Initial laboratory data are summarized in Table 1.

**Table 1:** Initial blood work.

Parameters	Patient values	Reference range
White blood cells	17.09 k/µL	4.5–11.0 k/µL
Hemoglobin	10.5 mg/dL	12–16 g/dL
Sodium	138 mEq/L	136–145 mEq/L
Chloride	118 mEq/L	98–107 mEq/L
Potassium	2.6 mEq/L	3.5–5.5 mEq/L
Bicarbonate	<5 mEq/L	22–29 mEq/L; baseline = 16–20 mEq/L
Anion gap	>15 mEq/L	4–12 mEq/L
Blood urea nitrogen	116 mg/dL	10–20 mg/dL; baseline = 40–50 mg/dL
Creatinine	8.56 mg/dL	0.55–1.11 mg/dL
Calcium	7.18 mg/dL	8.4–10.2 mg/dL
Albumin	2.4 g/dL	3.5–5.2 g/dL
Calculated osmolarity	328 mOsm/kg	275–295 mOsm/kg
Venous blood gas pH		
pH	7.04	7.33–7.43
PO_2_	32 mmHg	35–50 mmHg
PCO_2_	16 mmHg	38–50 mmHg

**Figure 1:**
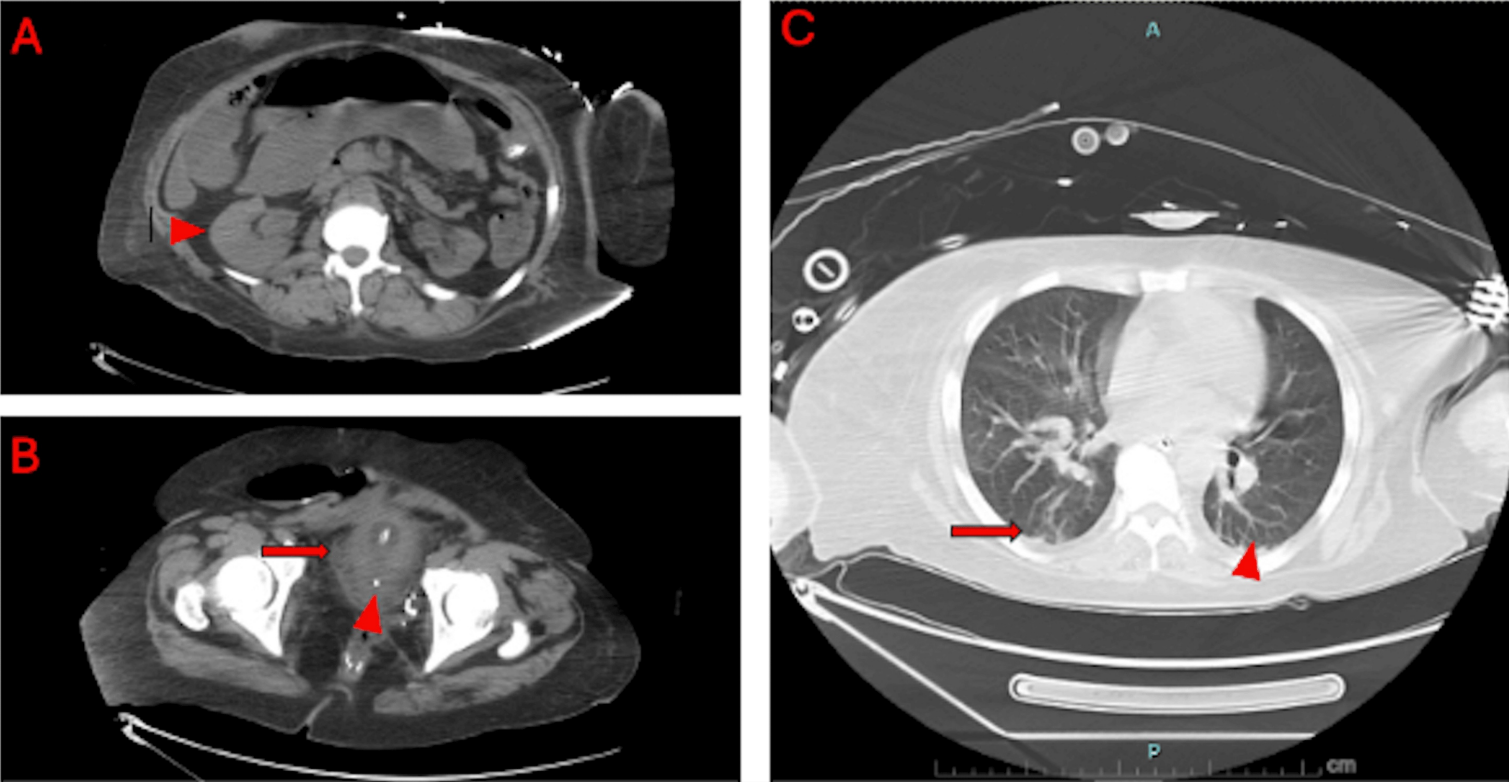
Axial CT scan of the abdomen and pelvis without contrast. (A) Left pyelitis (arrowhead). (B) Bladder calculi (arrowhead) and bladder fat stranding (arrow). (C) Lower lung opacities (arrow) and venous congestion (arrowhead).

**Figure 2:**
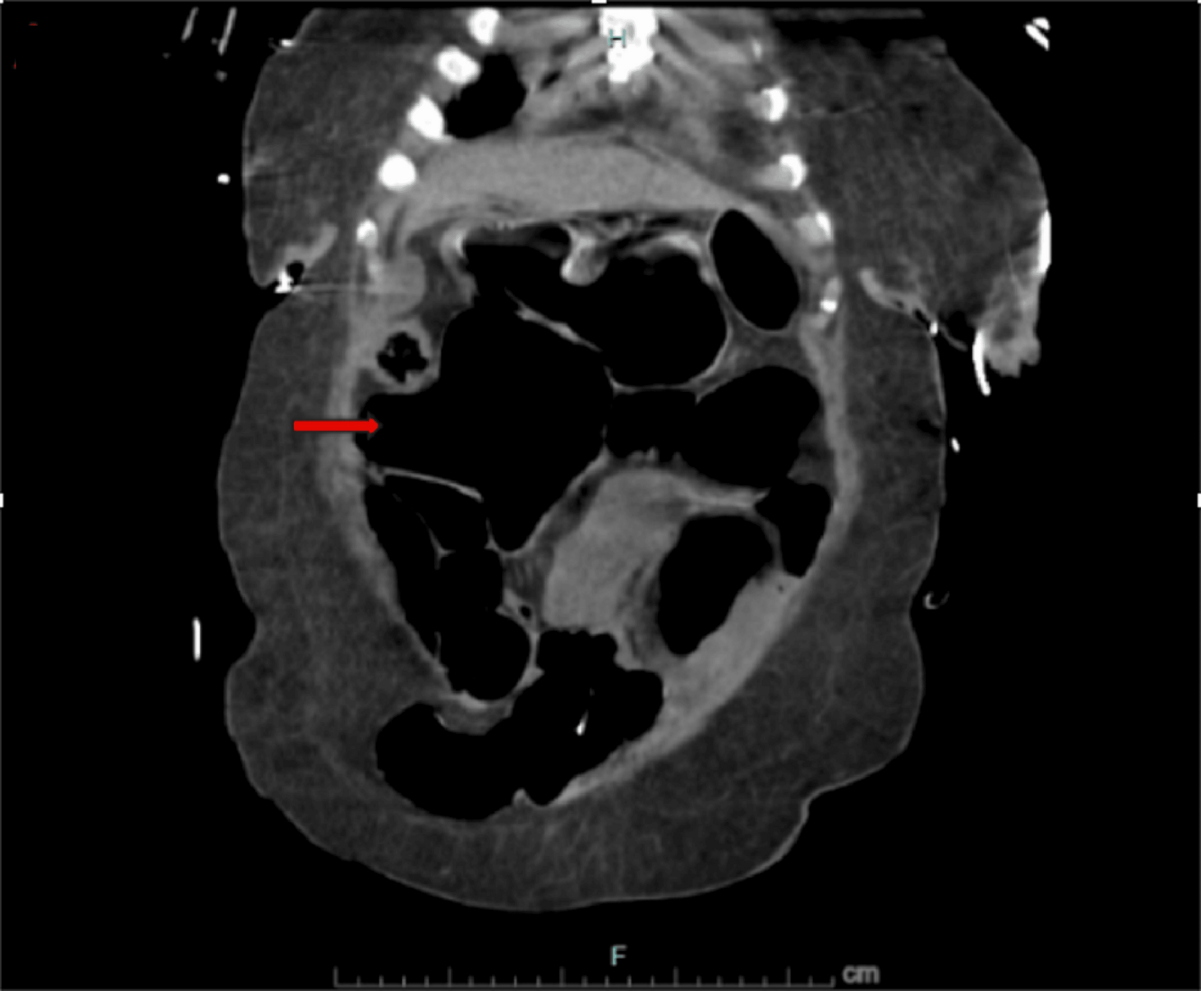
Coronal CT scan of the abdomen and pelvis without contrast. Air-fluid levels (arrow) suggestive of intestinal obstruction.

Lactic acid levels were within normal limits. Ammonia levels were not obtained. Urine toxicology screening was negative for opiates, fentanyl, benzodiazepines, barbiturates, cannabinoids, amphetamines, cocaine, and phencyclidine. Urinalysis was notable for proteinuria, hematuria, and pyuria, without the presence of urinary crystals. Electrocardiography revealed a normal sinus rhythm with no evidence of hypokalemia-related changes. Chest radiography showed no pleural effusion or consolidation; however, vascular cephalization was observed, suggestive of early pulmonary congestion. A non-contrast abdominal CT scan (Figures 1, 2) demonstrated findings consistent with cystitis, bilateral pyelitis, a bladder calculus, peri-vesical fat stranding, and air-fluid levels, raising concern for possible intestinal obstruction. Bilateral lower lung opacities were also noted.

The patient was admitted for management of urosepsis and initiated on a sodium bicarbonate infusion (150 mEq in sterile water), intravenous linezolid 600 mg every 12 hours, and piperacillin-tazobactam 2.25 g every 8 hours, along with potassium and magnesium repletion. Despite four hours of medical therapy, the patient’s tachypnea worsened, with her respiratory rate increasing to 32 breaths per minute. Given the persistent severe metabolic acidosis and escalating respiratory distress, a decision was made to initiate RRT for the first time, aiming to correct the acidosis and reduce the patient’s respiratory drive. The prescribed dialysis duration was 1.5 hours. The dialysate composition was as follows: sodium 137 mEq/L, potassium 4.0 mEq/L, calcium 3.0 mEq/L, magnesium 0.75 mEq/L, chloride 107.75 mEq/L, bicarbonate 33 mEq/L, acetate 4.0 mEq/L, and dextrose 200 mg/dL. Near the end of the dialysis session, the patient developed non-bloody emesis, hypotension, and transient hypoxia. Hemodialysis was discontinued after 80 minutes of treatment. Blood and dialysate flow rates were 150 mL/minute and 500 mL/minute, respectively. An F180NR high-flux dialyzer (surface area = 1.7 m², ultrafiltration coefficient (Kuf) = 76) was utilized. A total of 500 mL of blood volume was returned to the patient, resulting in a net positive fluid balance for the session. Blood glucose level at that time was 136 mg/dL. The patient was subsequently intubated and admitted to the intensive care unit (ICU) with vasopressor support. Post-dialysis laboratory values are summarized in Table 2.

**Table 2:** Post-dialysis chemistry parameters.

Parameters	Patient values	Reference range
Sodium	138 mEq/L	136–145 mEq/L
Potassium	2.0 mEq/L	3.5–5.5 mEq/L
Chloride	107 mEq/L	98–107 mEq/L
Bicarbonate	15 mEq/L	22–29 mEq/L
Anion gap	16 mEq/L	4–12 mEq/L
Blood urea nitrogen	49 mg/dL	10–20 mg/dL
Creatinine	3.41 mg/dL	0.55–1.11 mg/dL
Osmolality (calculated)	305 mOsm/kg	275–295 mOsm/kg

**Figure 3:**
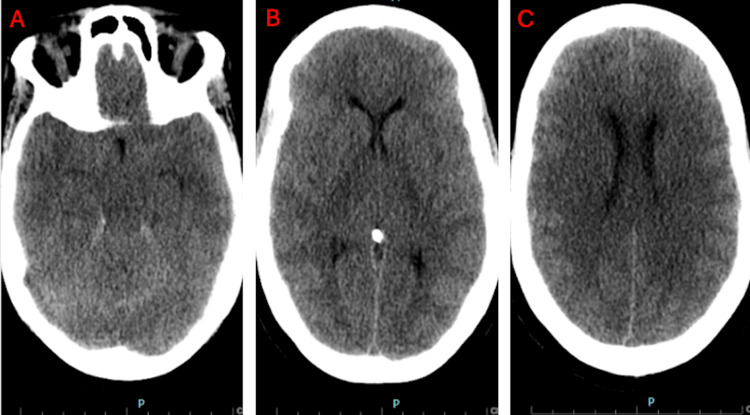
Axial CT scan of the head without contrast. (A-C) Caudal to cranial sequence demonstrating diffuse brain edema.

Shortly after arrival in the ICU, the patient was noted to have bilateral pupil dilation, absence of spontaneous movements, and absent brainstem reflexes. Due to suspected increased intracranial pressure, an immediate head CT scan (Figure 3) was performed, revealing mild generalized cerebral edema without hemorrhage.

**Figure 4:**
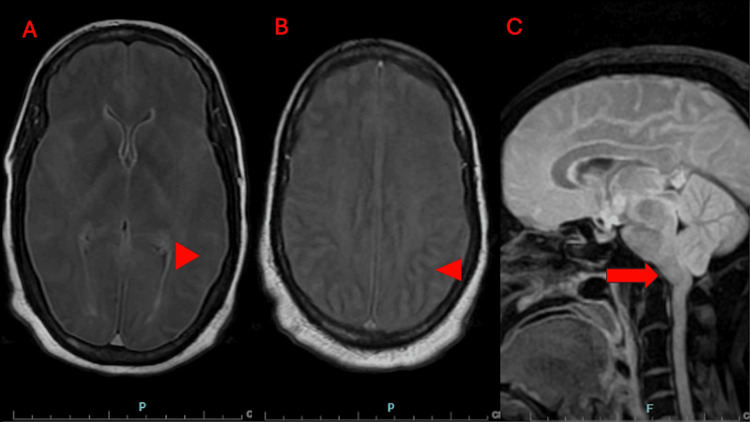
MRI of the brain without contrast. (A, B) Caudal to cranial sequence demonstrating diffuse abnormal signal throughout and sulcal effacement (arrowhead). (C) Mild transtentorial and cerebellar tonsillar herniation (arrow).

**Figure 5:**
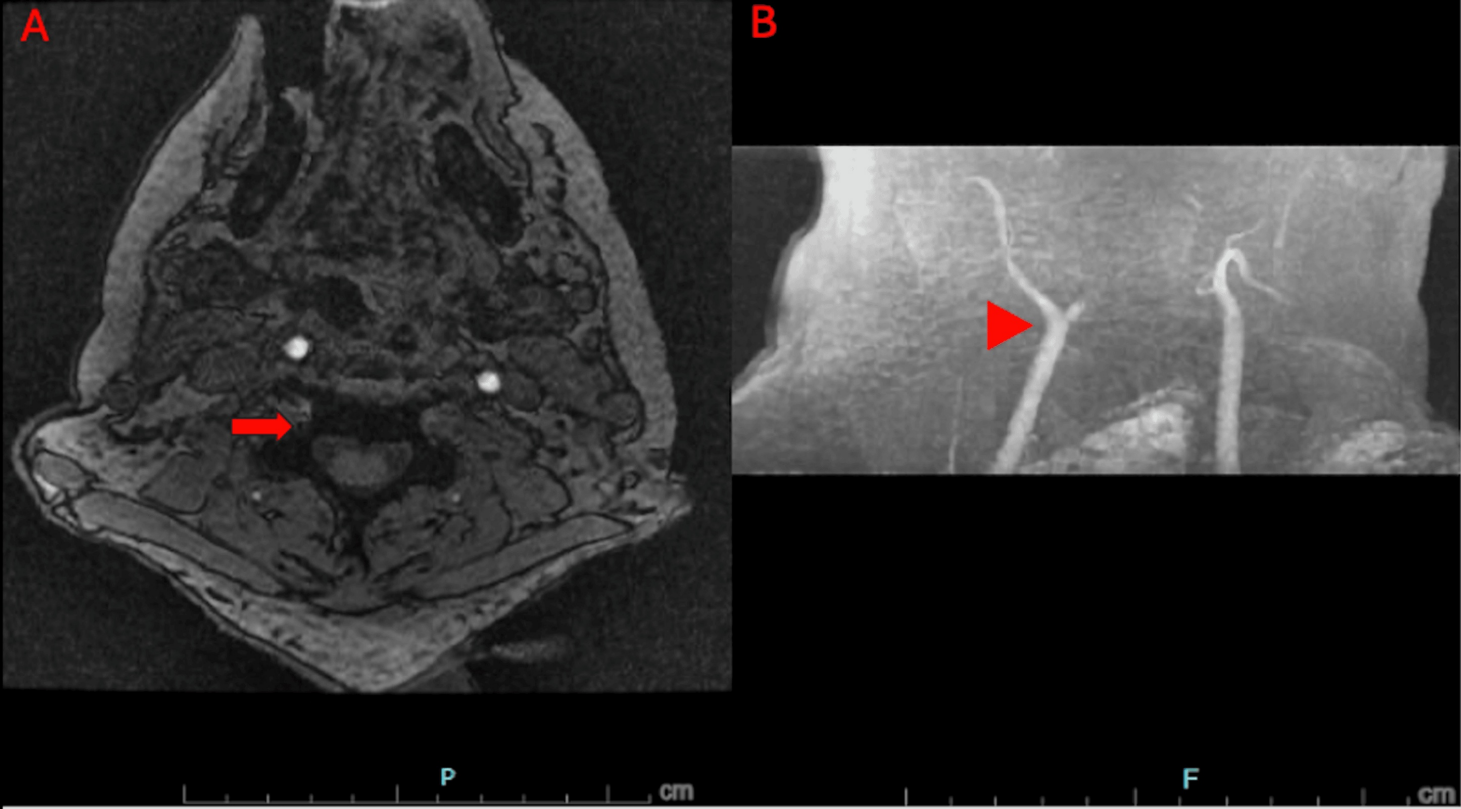
Time-of-flight MR angiography of the neck without contrast. (A) Absent vertebral artery signal (arrow). (B) No definite flow-related signal within the internal carotid arteries shortly after the bifurcations (arrowhead).

Alternate differential diagnoses for cerebral edema were considered, including acute stroke and DDS. Based on the presumptive diagnosis of DDS, the patient was treated with 500 mL of 3% hypertonic sodium chloride, 72 g of intravenous mannitol, head elevation, and initiation of a hyperventilation protocol to reduce cerebral swelling. Subsequent brain and neck MRI revealed global brain infarction, transtentorial and cerebellar tonsillar herniation, and compromised flow in both the anterior and posterior circulations (Figures 4, 5).

Despite treatment aimed at reversing herniation and the overall poor prognosis for meaningful recovery, the patient’s family elected to proceed with organ procurement. Evidence of brain herniation was observed 11 hours after arrival at the emergency department.

## Discussion

DDS was first described in the 1960s [12,13]. Symptoms are non-specific (Table 3) and may overlap with other diagnoses.

**Table 3:** Common symptoms of dialysis disequilibrium syndrome (DDS).

DDS	
Symptoms	Nausea, emesis, headache, dizziness, muscle cramps, agitation, disorientation, confusion, tremors, visual disturbances
Signs	Changes in mental status, asterixis, seizures, coma, death

It is important to recognize that changes in the brain after dialysis have been previously described and are not limited to DDS [14]. Most cases reported in the literature were identified by the presence of neurological symptoms and were commonly non-fatal [3]. Patients at risk for DDS include those with end-stage renal disease undergoing their first dialysis session, pediatric and elderly patients, and those with blood urea nitrogen (BUN) levels greater than 150 mg/dL, hypernatremia, hyperglycemia, metabolic acidosis, pre-existing neurological disorders, cerebral edema, or any condition that increases blood-brain barrier permeability [8]. Three main mechanisms are considered to contribute to the pathogenesis of DDS, namely, the reverse urea effect, the generation of intracellular osmoles, and the paradoxical brain acidosis effect. Under normal physiology, urea equilibrates slowly across body compartments (sieving coefficient = 0.44), limiting total water shifting, and thus is considered an ineffective osmole. Animal models have demonstrated that large interval changes in urea concentration between extracellular and intracellular compartments lead to a transient effective osmotic gradient capable of generating osmotic shifts [4]. This effect is further supported by the upregulation of aquaporins and downregulation of urea channels in brain cells during azotemia [5]. Furthermore, to prevent cell dehydration, brain cells adapt to changes in osmolality by generating intracellular amino acids, polyols, and trimethylamines known as “idiogenic” osmoles [6,7]. These osmoles protect cells from osmotic stress and are present in patients with chronic azotemia. However, they are slow to downregulate during sudden changes in brain osmolarity, leading to water influx and cellular edema. The third contributing factor to DDS, and the focus of our discussion, is the paradoxical intracellular brain acidosis that occurs during dialysis. This phenomenon follows rapid normalization of serum pH, causing blunting of compensatory mechanisms seen during metabolic acidosis, such as hyperventilation and increased carbonic anhydrase activity [7]. The result is increased carbon dioxide levels within the cerebrospinal fluid, causing displacement of bound intracellular cations by excess free hydrogen ions and promoting cellular swelling.

Current preventive strategies for DDS include short, slow dialysis sessions lasting less than two hours, with blood flow rates of 150-250 mL/minute and a target URR of less than 45% [8-11]. These approaches are based on expert consensus, as no official guidelines currently exist. The lack of robust evidence on DDS complicates establishing a definitive URR target; expert recommendations largely rely on case reports and fundamental understanding of urea kinetics [15]. In our case, the patient had a URR of 57% despite a short, slow dialysis prescription, which was a primary risk factor for DDS, although initial BUN levels were not as elevated compared to cases reported in the literature with fatal outcomes [1,2]. Additionally, hyperammonemia was considered but ruled out due to the absence of liver disease history, and no ammonia levels were obtained. The patient remained hypokalemic post-dialysis, likely due to concomitant bicarbonate infusion. It is important to note that hypokalemia has recently been associated with cerebral edema risk, especially in diabetic ketoacidosis patients, but it is not a traditional risk factor for DDS. Although hypokalemia may have contributed to cerebral swelling, the patient was dialyzed against a 4 mEq/L potassium bath, making it an unlikely major factor. Our case involved a bicarbonate correction exceeding 10 mEq/L. Experimental studies have demonstrated increased risk of cerebral edema with correction of metabolic acidosis during serum alkalinization, including animal studies in which higher intracranial pressures were observed when dialyzed against high bicarbonate baths [16]. A prospective study has also demonstrated a low risk of acidosis overcorrection when decreasing bicarbonate concentration in dialysate [17]. In pediatric populations, Glaser and colleagues have documented cerebral edema risk with serum alkalinization [18]. No similar studies exist in adult populations examining the paradoxical brain acidosis effect.

We hypothesize that the degree of acidosis correction in our patient accelerated DDS development despite a BUN below 150 mg/dL and a reasonable renal replacement therapy flow prescription. This is supported by the patient’s abrupt worsening hypoxia, likely due to the leftward shift of the oxyhemoglobin dissociation curve seen during serum alkalinization [19]. This shift decreases hemoglobin’s oxygen release, potentially leading to diffuse tissue hypoxia and inflammation. Notably, the oxyhemoglobin dissociation curve in CKD patients with acidemia is typically shifted rightward, facilitating oxygen release, a phenomenon exacerbated by anemia, which was also present in our patient. The timing of fatal DDS development varies; once suspected, treatment aims to prevent brain injury and alleviate symptoms. Therapies reported in the literature are mostly anecdotal and focus on reducing intracranial pressure using hyperosmolar agents such as hypertonic saline and mannitol. The role of hyperventilation remains unclear due to a lack of research. Unfortunately, optimal treatment protocols remain unknown and are a subject of ongoing debate.

## Conclusions

Pre-dialysis metabolic acidosis is a significant risk factor for fatal outcomes in DDS, and gradual correction should be considered when developing preventive strategies. While correcting profound acidemia before initiating dialysis may be ideal, it is often limited by the patient’s clinical status and risk of rapid deterioration. In such cases, a slow form of dialysis called continuous RRT may be the preferred modality. However, further research is needed to establish definitive guidelines. Additional studies are also necessary to better understand the role of metabolic acidosis in the development of cerebral edema.
